# Impaired activity of CCA-adding enzyme TRNT1 impacts OXPHOS complexes and cellular respiration in SIFD patient-derived fibroblasts

**DOI:** 10.1186/s13023-016-0466-3

**Published:** 2016-06-18

**Authors:** Urszula Liwak-Muir, Hapsatou Mamady, Turaya Naas, Quinlan Wylie, Skye McBride, Matthew Lines, Jean Michaud, Stephen D. Baird, Pranesh K. Chakraborty, Martin Holcik

**Affiliations:** Molecular Biomedicine Program, Children’s Hospital of Eastern Ontario Research Institute, 401 Smyth Road, Ottawa, ON K1H 8L1 Canada; Department of Pediatrics, University of Ottawa, Ottawa, Ontario K1H 8M5 Canada; Newborn Screening Ontario, Children’s Hospital of Eastern Ontario, Ottawa, ON Canada; Department of Pathology and Laboratory Medicine, Children’s Hospital of Eastern Ontario and University of Ottawa, Ottawa, Ontario Canada

**Keywords:** SIFD, TRNT1, OXPHOS, Mitochondria, Translation

## Abstract

**Background:**

SIFD (Sideroblastic anemia with B-cell immunodeficiency, periodic fevers, and developmental delay) is a novel form of congenital sideroblastic anemia associated with B-cell immunodeficiency, periodic fevers, and developmental delay caused by mutations in the CCA-adding enzyme TRNT1, but the precise molecular pathophysiology is not known.

**Results:**

We show that the disease causing mutations in patient-derived fibroblasts do not affect subcellular localization of TRNT1 and show no gross morphological differences when compared to control cells. Analysis of cellular respiration and oxidative phosphorylation (OXPHOS) complexes demonstrates that both basal and maximal respiration rates are decreased in patient cells, which may be attributed to an observed decrease in the abundance of select proteins of the OXPHOS complexes.

**Conclusions:**

Our data provides further insight into cellular pathophysiology of SIFD.

**Electronic supplementary material:**

The online version of this article (doi:10.1186/s13023-016-0466-3) contains supplementary material, which is available to authorized users.

## Background

The tRNA nucleotidyl transferase (*TRNT1*) gene encodes a protein involved in the maturation of all cytosolic and mitochondrial tRNAs. It functions to add CCA to the 3’ terminus of tRNA molecules and is required for proper aminoacylation of all tRNAs [1]. We recently described a cohort of patients with congenital sideroblastic anemia with B-cell immunodeficiency, periodic fevers and developmental delay (SIFD) [2] due to mutations in *TRNT1*. We demonstrated that patient mutations result in an impairment of TRNT1 to catalyze the addition of CCA to tRNA, while the knockdown of *TRNT1* results in significant cytotoxicity and apoptosis. It was shown subsequently that mutations in *TRNT1* correlate with the incomplete addition of CCA to the mitochondrial serine-tRNA (AGY;[3]), a tRNA possessing a non-canonical structure [4]. SIFD belongs to a group of congenital sideroblastic anemias (CSAs) that are characterized by iron deposits in the mitochondria of red blood cell precursors. Given that all CSA-associated genes identified to date are involved in mitochondrial pathways [5], and the critical role that TRNT1 plays in mitochondrial tRNA maturation, we sought to determine the effect of mutated *TRNT1* on patient mitochondrial function.

## Methods

### Cell culture

Control fibroblasts were obtained from Coriell Institute (Control 1, Cat# GM08680; Control 2, Cat# GM00498) and from a healthy female donor (Control 3). Patient fibroblasts were previously described; patient 7 is compound heterozygous TRNT1 p.L166S, p.T154I, whereas patient 8 is homozygous for the TRNT1 p.R190I mutation [2]. Cells were maintained in Dulbecco's modified Eagle's medium (DMEM, Thermo Scientific) supplemented with heat-inactivated 10 % fetal calf serum, 2 mM L-glutamine, and 1 % antibiotics (100 units/ml penicillin–streptomycin) at 37 °C and 5 % CO_2_.

### Cytoplasmic translation assay

Metabolic labeling was done as previously described [6]. Briefly, control and patient-derived fibroblasts were grown in 6-well plates, washed twice in 1 mL of DMEM lacking methionine and cysteine (DMEM-Met,Cys) supplemented with 10 % fetal calf serum and 2 mM L-glutamine and incubated in 1 mL of DMEM-Met,Cys for 15 minutes at 37 °C and 5 % CO_2_. 1 mL of 100 μCi ^35^S-Met, Cys (Perkin Elmer; NEG772) was added and the cells were incubated for an additional 25 minutes at 37 °C. Cells were washed three times in cold PBS (300 x g/5 min/4 °C) and lysed in RIPA (50 mM Tris [pH7.4], 150 mM NaCl, 1 mM EDTA, 0.25 % sodium deoxycholate, 1 % Igepal, protease inhibitors) buffer. Total protein content was quantified by Bradford protein assay (BioRad) and equal amounts of proteins were separated by 10 % SDS-PAGE, stained with Coomassie R-250 dye, and exposed to X-ray film.

### Western blotting

Cells were washed in 1 mL of PBS and lysed in 150 μL of RIPA buffer for 30 minutes at 4 °C, followed by centrifugation at 12,000 x g for 10 minutes to pellet debris. Protein concentrations were determined by Bradford protein assay, and equal amounts of protein extract were separated by 10 % SDS-PAGE and transferred to PVDF membrane. Proteins were identified using anti-OXPHOS Human cocktail (Abcam; ab110411), anti-Tom20 (Santa Cruz Biotechnology; sc-11415), anti-TRNT1 (Novus Biologicals; NBPI-86589) or anti-Tubulin (Abcam; ab7291) followed by species-specific HRP-conjugated secondary antibodies (Cell Signaling Technology). Antibody complexes were detected using an ECL system (GE Biosciences) and the densitometry analyses were performed using the ImageJ software.

### Immunofluorescence

Fibroblasts were cultured on coverslips or in 384-well plates (BD Falcon) for 24 hours in DMEM. Cells were fixed with 3.7 % formaldehyde at room temperature for 15 minutes, washed twice with PBS, and permeabilized for 15 minutes with 0.1 % Triton X-100 (Sigma) in PBS. Cells were blocked with 3 % bovine serum albumin (Invitrogen) for 30 minutes and then incubated overnight at 4 °C with rabbit anti-TRNT1 primary antibody (Novus Biologicals) diluted 1/50 in PBS buffer containing 2 % BSA and 0.004 % Triton X-100, or mouse anti-TOM20 primary antibody diluted 1/500. Cells were washed and incubated with AlexaFluor-488 anti-rabbit or AlexaFluor-594 anti-mouse antibodies for 1 hour at room temperature. Nuclei were stained with 2 μg/mL Hoechst 33342 (Sigma). Cells were imaged using the Olympus Fluoview FV-1000 Laser Confocal Microscope or using the Opera Imaging system and analyzed using the Columbus Image Analysis Software and Acapella image analysis scripting language (Perkin Elmer).

### Mitochondrial membrane potential staining

Measurement of mitochondrial membrane potential was determined using the potential-sensitive dye Tetramethylrhodamine ethyl ester perchlorate (TMRE; Molecular Probes). Briefly, 2000-3000 cells were cultured in a 384-well microplate for 48 hours then incubated for 30 minutes in DMEM containing 20 nM MitoTracker Green FM (Molecular Probes). The cells were pre-incubated with 20 μM Carbonyl cyanide *m*-chlorophenyl hydrazone (CCCP; to eliminate mitochondrial membrane potential) or solvent control at 37 °C for 1 hour prior to adding TMRE. After washing with media, 25 nM TMRE in media containing 0.5 μg/mL Hoechst 33342 (Molecular Probes) was added to the cells and incubated for 1 hour at 37 °C. TMRE and MitoTracker Green fluorescence intensities of the mitochondria in live cells were imaged using the Opera automated confocal microscope and analyzed with the Columbus Image Analysis Software (Perkin Elmer).

### Micro-oximetry analysis

Oxygen consumption rate (OCR) was measured using the XF24 Extracellular Flux Analyzer (Seahorse Bioscience). Control and patient fibroblasts were seeded in XF 24-well cell culture microplates at 4 x10^4^ cells/well and incubated at 37 °C/5 % CO_2_ for 24 hours. The XF Assay Cartridge was calibrated by adding XF calibrant for 18 hours in a 37 °C - CO_2_ free incubator. During the equilibration period, the XF24 Analyzer was calibrated with a calibration plate using the standard XF calibration protocol. The assay was initiated by replacing the growth medium from each well with 600 μL of bicarbonate free/unbuffered DMEM-based media (XF assay medium) containing 25 mM glucose and 1 mM sodium pyruvate, pre-warmed at 37 °C. The cells were incubated at 37 °C for 1 hour in a CO_2_ free incubator to allow media temperature and pH to reach equilibrium before the first measurement. XF cell Mito Stress Test Kit reagents were used to measure mitochondrial respiration. The drug injections ports of the XF assay cartridge were loaded with the concentrated assay reagents (13X stock) in the assay medium. After three measurements of the baseline OCR, 10 μM oligomycin, 7 μM carbonyl cyanide 4-(trifluoromethoxy) phenylhydrazone (FCCP), 5 μM antimycin A and 5 μM rotenone were sequentially added to each well. The changes in the OCR measurements were analyzed and the data expressed as pmol of O_2_ per minute. The data were normalized by the number of cells determined by Vybrant Green DNA stain (Life Technologies, Invitrogen) at 1 μM final concentration. Dye incorporation was measured using the INCUCYTE™ ZOOM live-cell imaging System (Essen Bioscience).

### Transmission electron microscopy

1 x 10^6^ cells were fixed in 2.5 % cacodylate buffered glutaraldehyde for a minimum of 2 hours. Cells were centrifuged at 1800 x g for 10 minutes and the pellet resuspended in 2 mL sodium cacodylate buffer, pH 7.2. Through subsequent pelleting and resuspension, cells were post-fixed in 2 % osmium tetroxide for 2 hours, rinsed in water, and dehydrated through graded ethanol up to absolute. A final dehydration in pure acetone was followed by three changes in Spurr’s resin (Canemco) and a final embedding at 65 °C. Thin (80 nm) sections were cut using an Ultracut R ultramicrotome (Leica) and stained with uranyl acetate, and lead citrate. Grids were screened on a Hitachi 7100 transmission EM and the images digitally captured. Images for 10 cells per sample were captured at low (3000X) and high (30,000X) magnification. The number of mitochondria per cell were counted manually using ImageJ software.

### Statistical analysis

Statistical analysis was performed using GraphPad Prism version 6.00 for Windows (GraphPad Software, San Diego, CA) to determine *p*-value in repeated experiments, and the type of test is indicated in the Figure legend. All results are shown as mean +/- standard deviation. Unless otherwise noted, all results were obtained through a minimum of three independent experimental replications.

## Results

**Fig. 1:**
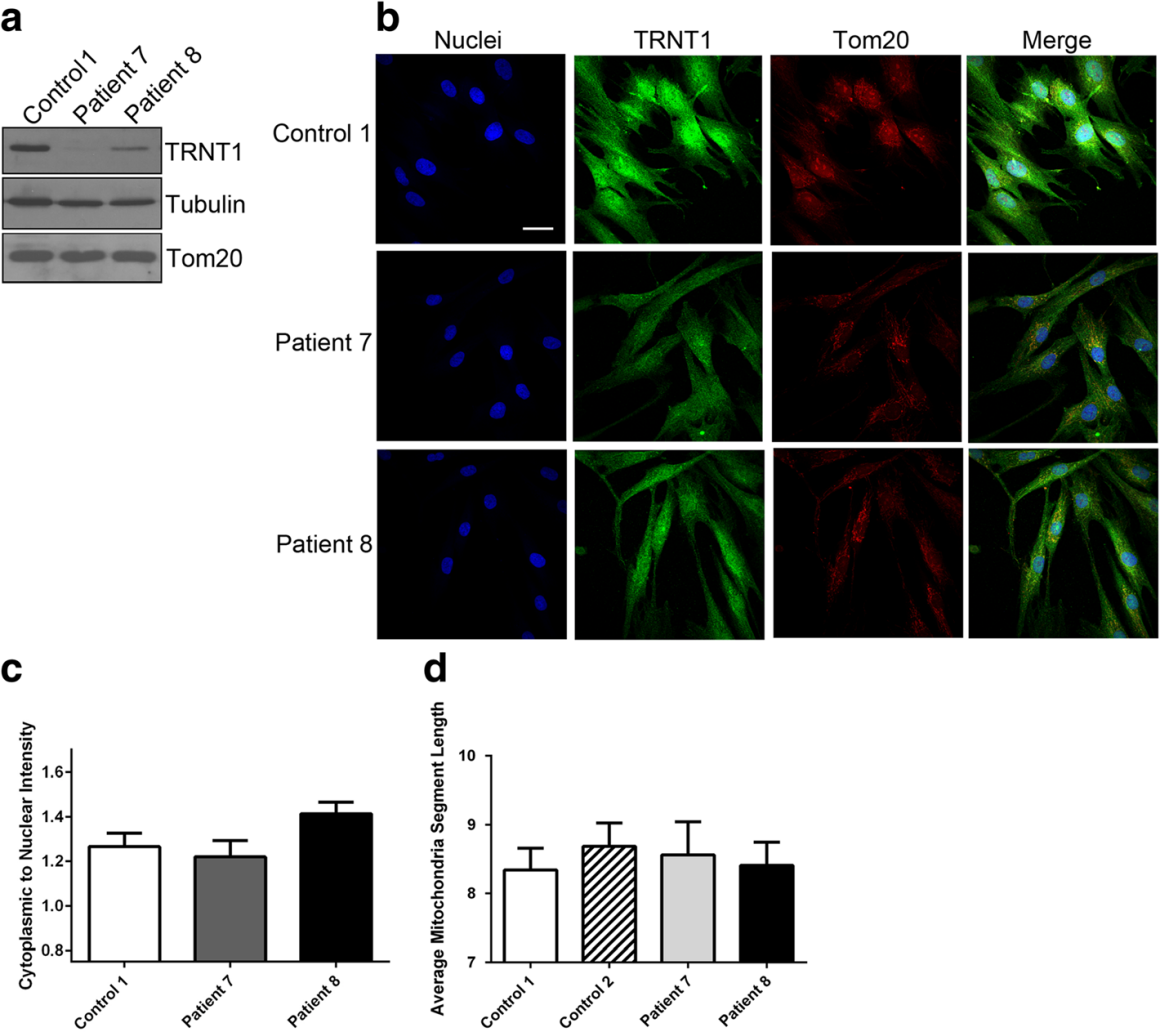
Expression and localization of TRNT1 in patient-derived fibroblasts. **a** TRNT1 protein expression was analyzed in patient and control fibroblasts by western blot analysis. Tubulin and Tom20 were used as loading controls. **b** Immunofluorescent imaging of TRNT1 in fibroblast cells. Nuclei are identified by Hoechst stain and mitochondria is stained with an anti-Tom20 antibody (bar = 20 μm). **c** The expression of TRNT1 in the cytoplasm and nucleus was calculated from images obtained on the Opera automatic confocal microscope. The ratio of the average intensity for TRNT1 signal in the cytoplasm to nucleus was measured in approximately 90 cells per well. The mean ratio per well was averaged over 6 wells and the standard deviation was calculated from the mean ratio values of the 6 wells

**Fig. 2:**
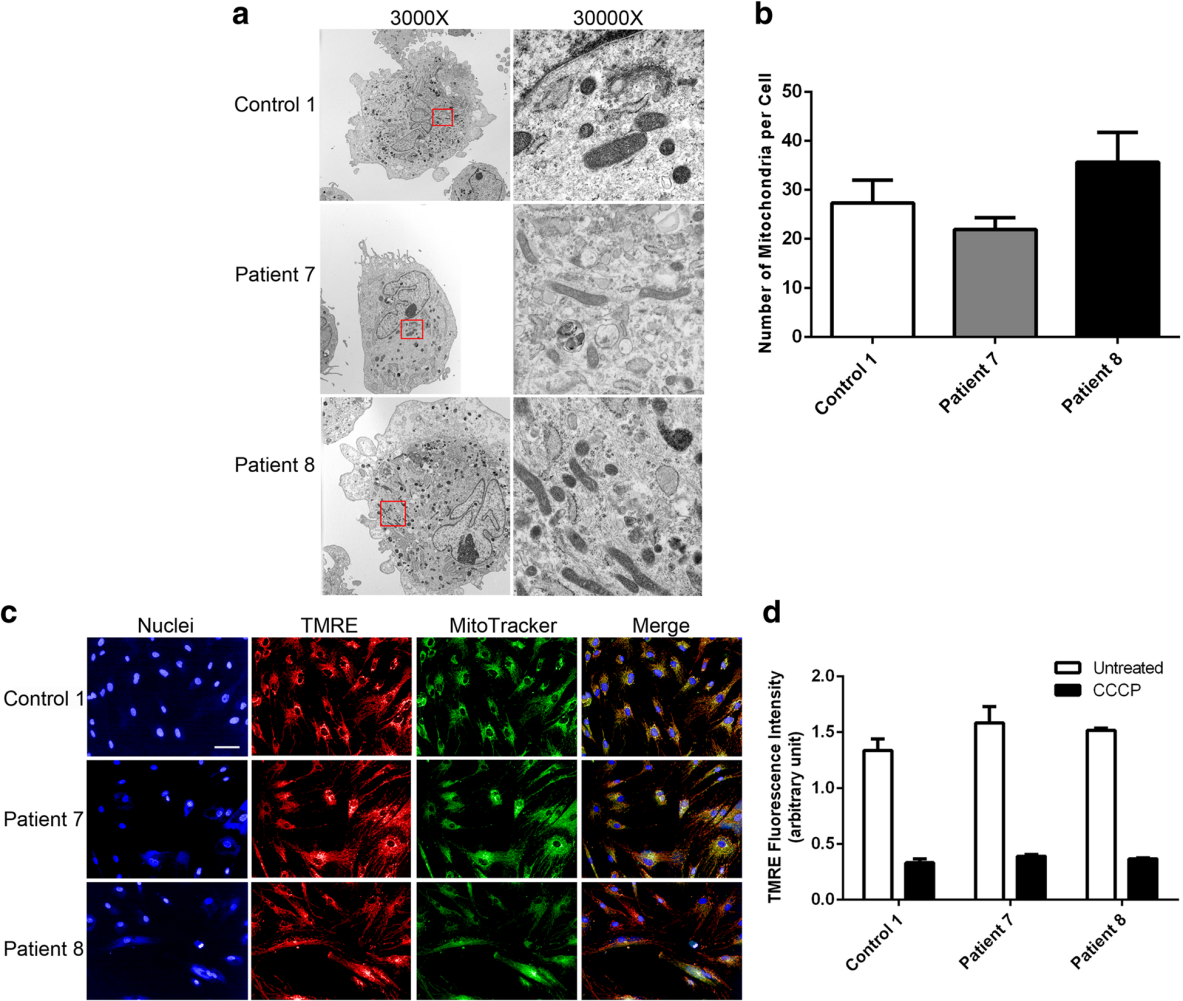
Mitochondria are not altered in patient-derived fibroblasts. **a** Electron microscopy images of control and patient-derived fibroblasts at 3000X and 30,000X. The red box identifies the area under 30,000X magnification. **b** The number of mitochondria was determined using ImageJ software by manual counting from 10 cells each from control or patients from the EM images. **c** Immunofluorescence images of the mitochondrial membrane potential as monitored by the incorporation of the TMRE dye and compared to the total mitochondrial stain as measured by MitoTracker. Nuclei are identified by Hoechst stain. (bar = 50 μm) (**d**) The relative TMRE intensity was measured and compared to cells treated with the uncoupler CCCP

We used fibroblasts derived from patients and healthy controls for analysis. Patient 7 is compound heterozygous for TRNT1 p.L166S and p.T154I mutations, whereas patient 8 is homozygous for the TRNT1 p.R190I mutation [2]. We first examined by western blot analysis the levels of TRNT1 in patient-derived and control fibroblasts and confirmed a decrease in the expression of TRNT1 in patient cells (Fig. 1a). We next investigated whether the patient-derived mutations alter the localization of TRNT1. We visualized localization of endogenous TRNT1 by immunofluorescence and counterstained cells with a Tom20 antibody (to identify the mitochondria) and Hoechst stain (to identify nuclei) (Fig. 1b). We observed that TRNT1 in patient-derived fibroblasts localizes to the nucleus, cytosol, and mitochondria similar to control. Furthermore, although the expression levels of TRNT1 are reduced in patient cells, the ratio of cytoplasmic to nuclear TRNT1 did not differ significantly, suggesting that the mutations in TRNT1 do not affect the subcellular localization of TRNT1 (Fig. 1c). Mitochondria undergo dynamic fusion and fission and the extent of the fused network may have an effect on cell viability [7]. To determine if there is a defect in fission or fusion, we stained the cells with a Tom20 antibody and measured the length to width ratio of the mitochondrial network (Additional file 1: Figure S1; [8]). We did not observe a significant difference in the mitochondrial network between patient-derived and control fibroblasts (Fig. 1d). The cellular ultrastructure of patient and control fibroblast cells was further examined by electron microscopy (Fig. 2a and Additional file 1: Figure S2) and we did not observe any gross morphological differences in mitochondria from patient or control cells. Furthermore, we counted the number of mitochondria per cell and found no significant difference between patients and control (Fig. 2b). Next, to determine any changes in the mitochondrial transmembrane potential in patient cells as compared to control we treated cells with TMRE (to monitor membrane potential) and with MitoTracker Green (to stain all mitochondria regardless of potential) (Fig. 2c and Additional file 1: Figure S3) and quantified the relative TMRE fluorescence as compared to fluorescence with the uncoupler CCCP (Fig. 2d). We determined that there is no significant difference in mitochondrial membrane potential in patient-derived fibroblasts when compared to control cells.

**Fig. 3:**
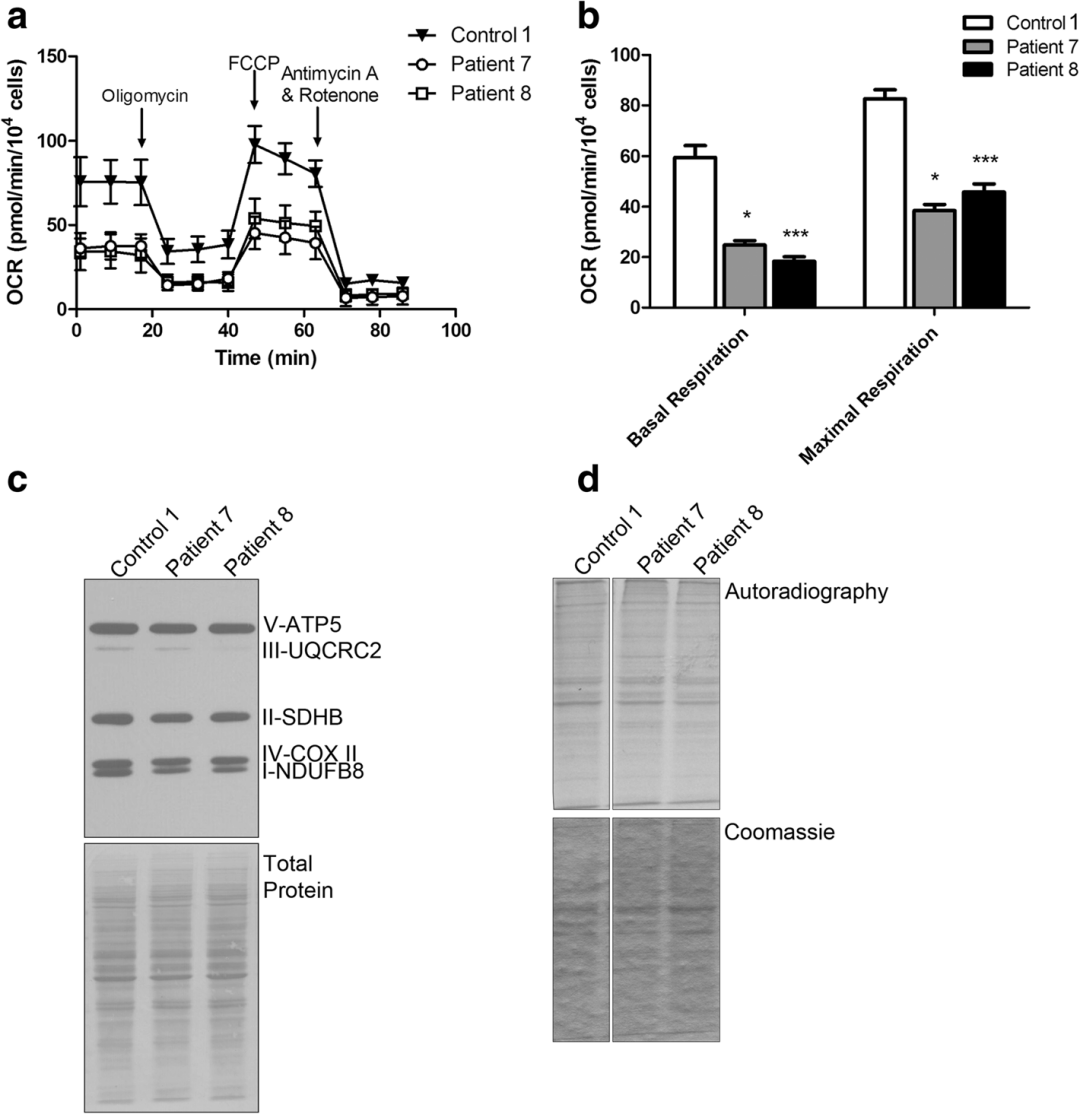
Decreased oxygen consumption rates correlate with a loss in OXPHOS complex protein expressions. **a** Oxygen consumption rate (OCR) of patient and control fibroblasts as measured by micro-oximetry analysis. **b** Basal and maximal OCR was calculated from repeated experiments (*p < 0.05, ***p < 0.0001; Kruskal-Wallis test with a Dunn's multiple comparisons post test) (**c**) Western blot analysis of 5 OXPHOS proteins representing each of the complexes involved in oxidative phosphorylation. **d** ^35^S-methionine incorporation assay to measure global cytoplasmic translation. Autoradiography represents the ^35^S signal from newly synthesized proteins and Coomassie represents total protein loading

Next, we examined mitochondrial respiration by micro-oximetry to assess any differences in mitochondrial oxidative phosphorylation (OXPHOS) in patient fibroblasts compared to that of the controls (Fig. 3a). We observed a significant difference in the overall basal and maximal oxygen consumption rates (OCR; Fig. 3b) in patient cells when compared to control. We therefore examined the expression levels of some of the proteins of the OXPHOS complexes by western blot analysis (Fig. 3c and Additional file 1: Figure S4). We used an antibody cocktail that probes for representative proteins of the OXPHOS complexes as these proteins are known to be labile when the complex is not properly assembled. Interestingly, we observed a decrease in NDUFB8 (complex I), SDHB (complex II), and COX II (complex IV) proteins. Since NDUFB8 and SDHB are encoded in the nucleus and translated in the cytoplasm, we performed ^35^S-methionine/cysteine incorporation assays in patient-derived fibroblasts (Fig. 3d) to determine if global translation defects could explain the loss in expression of these proteins. Surprisingly, we observed that global translation is not impaired in fibroblasts with mutated *TRNT1*.

## Discussion

We have recently described a novel form of congenital sideroblastic anemia that is associated with B-cell immunodeficiency, periodic fevers and developmental delay (SIFD) in which we identified mutations in *TRNT1* and demonstrated that these mutations are disease causing. We were interested in studying the mechanisms by which mutated *TRNT1* might cause disease. Since some of the symptoms associated with SIFD appear to be related to metabolic syndrome, we were interested in determining how the patient-derived *TRNT1* mutations affect mitochondrial biology, and we focused our studies on mitochondrial processes in patient-derived fibroblasts. Interestingly, immunofluorescence analysis of TRNT1 subcellular localization and electron microscopy of cells showed no gross morphological differences between patient and control cells, and that the number of mitochondria per cell and their structure are similar. Our initial findings using micro-oximetry analysis demonstrated that basal and maximal respiration are decreased in patient cells as compared to healthy control cells. Furthermore, our western blot analyses further suggest a defect in cellular respiration by demonstrating a decrease in expression of some OXPHOS complex subunits. Notably, at least two of the affected proteins (NDUFB8 (complex I) and SDHB (complex II)) are encoded in the nucleus, an observation which would be consistent with the global role of TRNT1 in tRNA maturation. However, we did not observe any defect in cytoplasmic translation, suggesting that the decrease in abundance of these proteins might be due to complex assembly, rather than protein synthesis. Because the OXPHOS system is assembled from both nuclear- and mitochondrial-encoded components, proper assembly of these complexes is essential and, in fact mutations in assembly factors are associated with human pathologies [9]. We have also observed a specific decrease in the abundance of the mitochondrial–encoded COX II (complex IV) protein. We are currently conducting SILAC experiments to identify the full spectrum of changes in the abundance and stoichiometry of all OXPHOS complexes as well as changes in the abundance of additional mitochondrial proteins.

Sasarman et al. (2015) have recently described a defect in mitochondrial tRNASer (AGY) CCA addition associated with *TRNT1* mutation that leads to impairment of mitochondrial translation and decreased abundance in 13 mitochondrial subunits of OXPHOS complexes, which is congruent with our observation. In contrast to our observations, however, the changes observed by Sasarman et al were restricted to muscle biopsies with minimal effect seen in fibroblasts. It is possible that this difference is due to distinct *TRNT1* mutations found in patients. Indeed, both patient 7 and patient 8 displayed both reduced TRNT1 activity [2] and expression levels (Fig. 1), presumably leading to more severe TRNT1 deficiency than that observed by Sasarman et al. Of note, both patient 7 and patient 8 are deceased suggesting that these mutations are on the more severe end of the TRNT1 pathology spectrum. Recently, hypomorphic mutations in *TRNT1* were also found to cause rare non-syndromic retinitis pigmentosa with erythrocytic microcytosis [10]. The three patients in this study displayed nyctalopia, microcytosis and anisocytosis, but were otherwise healthy and lacking typical SIFD symptoms such as developmental delay, deafness, ataxia, seizures or cardiac disease. In contrast to our patient cohort, the mutations found in these patients were located to the extreme C-terminus or the N-terminal head-domain of TRNT1 and could be considered to have a mild effect on TRNT1 function. Interestingly, however, morpholino-based modest reduction of TRNT1 levels in zebrafish model recapitulated the vision-selective phenotype, whereas the more severe knock-down also resulted in an eye and heart development abnormalities, reduced touch response and erythrocyte maturation, recapitulating some of the SIFD features. Furthermore, TRNT1 morphants displayed reduced CCA-incorporation efficiency similar to our patients. Unfortunately this study did not assess the mitochondrial activity in either patients or zebrafish model, Taken together these data further support the notion that the clinical variability associated with SIFD may be linked to distinct mutations in *TRNT1* and expression levels of TRNT1 resulting in an imbalance in cytoplasmic and mitochondrial protein synthesis and the consequent effect on mitochondrial function.

## Conclusions

We conclude that although patient fibroblast cells do not appear to be defective in cellular morphology, TRNT1 localization, global translation, mitochondrial network architecture, or mitochondrial transmembrane potential, patient cells harboring mutated *TRNT1* are defective in their ability to properly form OXPHOS complexes and consume oxygen. This likely contributes to the observed mitochondrial phenotypes associated with mutant *TRNT1* and SIFD.

## Abbreviations

CCCP, Carbonyl cyanide *m*-chlorophenyl hydrazone; DMEM, Dulbecco's modified Eagle's medium; OCR, Oxygen consumption rate; OXPHOS – oxidative phosphorylation; SIFD, Sideroblastic anemia with B-cell immunodeficiency, periodic fevers, and developmental delay; TRNT1, tRNA Nucleotidyl Transferase

## Additional file

Additional file 1: Figure S1. Immunofluorescent imaging of mitochondrial network in fibroblast cells. (Left) Nuclei are identified by Hoechst stain (red) and mitochondria are stained with an anti-Tom20 antibody (green). Representative images are shown. (Right) The mitochondria segment length was calculated as the pixel distance from between either the end of a mitochondria or a branch point or the crossing over of another mitochondria and is averaged for each cell using a custom Acapella script run in the Columbus software. From 100 to 200 cells were measured per well and averaged over 14 wells for three separate experiments. **Figure S2.** Electron microscopy images of additional control fibroblasts. The red box identifies the area enlarged on the right. **Figure S3.** Additional control fibroblasts for membrane potential assessment. Shown are immunofluorescence images of the mitochondrial membrane potential as monitored by the incorporation of the TMRE dye (red) and compared to the total mitochondrial stain as measured by MitoTracker (green). Nuclei are identified by Hoechst stain (blue).**Figure S4.** (A) Oxygen consumption rate (OCR) of three control fibroblasts as measured by micro-oximetry analysis. (B) Densitometry of western blot analysis of 5 OXPHOS proteins. The band intensities were determined using ImageJ software. The intensity of each band is shown relative to total protein (ns = not significant; * = *p* < 0.05; ** = p<0.01; student *t*-test). (PDF 2743 kb)
