# Supplementary material for: Impaired activity of CCA-adding enzyme TRNT1 impacts OXPHOS complexes and cellular respiration in SIFD patient-derived fibroblasts
Source: Orphanet J Rare Dis. 2016 Jun 18;11:79. doi: 10.1186/s13023-016-0466-3 (PMC4912790; doi:10.1186/s13023-016-0466-3)
Supplement: Additional file 1: Figure S1. — Immunofluorescent imaging of mitochondrial network in fibroblast cells. (Left) Nuclei are identified by Hoechst stain (red) and mitochondria are stained with an anti-Tom20 antibody (green). Representative images are shown. (Right) The mitochondria segment length was calculated as the pixel distance from between either the end of a mitochondria or a branch point or the crossing over of another mitochondria and is averaged for each cell using a custom Acapella script run in the Columbus software. From 100 to 200 cells were measured per well and averaged over 14 wells for three separate experiments. Figure S2. Electron microscopy images of additional control fibroblasts. The red box identifies the area enlarged on the right. Figure S3. Additional control fibroblasts for membrane potential assessment. Shown are immunofluorescence images of the mitochondrial membrane potential as monitored by the incorporation of the TMRE dye (red) and compared to the total mitochondrial stain as measured by MitoTracker (green). Nuclei are identified by Hoechst stain (blue).Figure S4. (A) Oxygen consumption rate (OCR) of three control fibroblasts as measured by micro-oximetry analysis. (B) Densitometry of western blot analysis of 5 OXPHOS proteins. The band intensities were determined using ImageJ software. The intensity of each band is shown relative to total protein (ns = not significant; * = p < 0.05; ** = p<0.01; student t-test). (PDF 2743 kb) [file 13023_2016_466_MOESM1_ESM.pdf]

Supplemental Figure 1

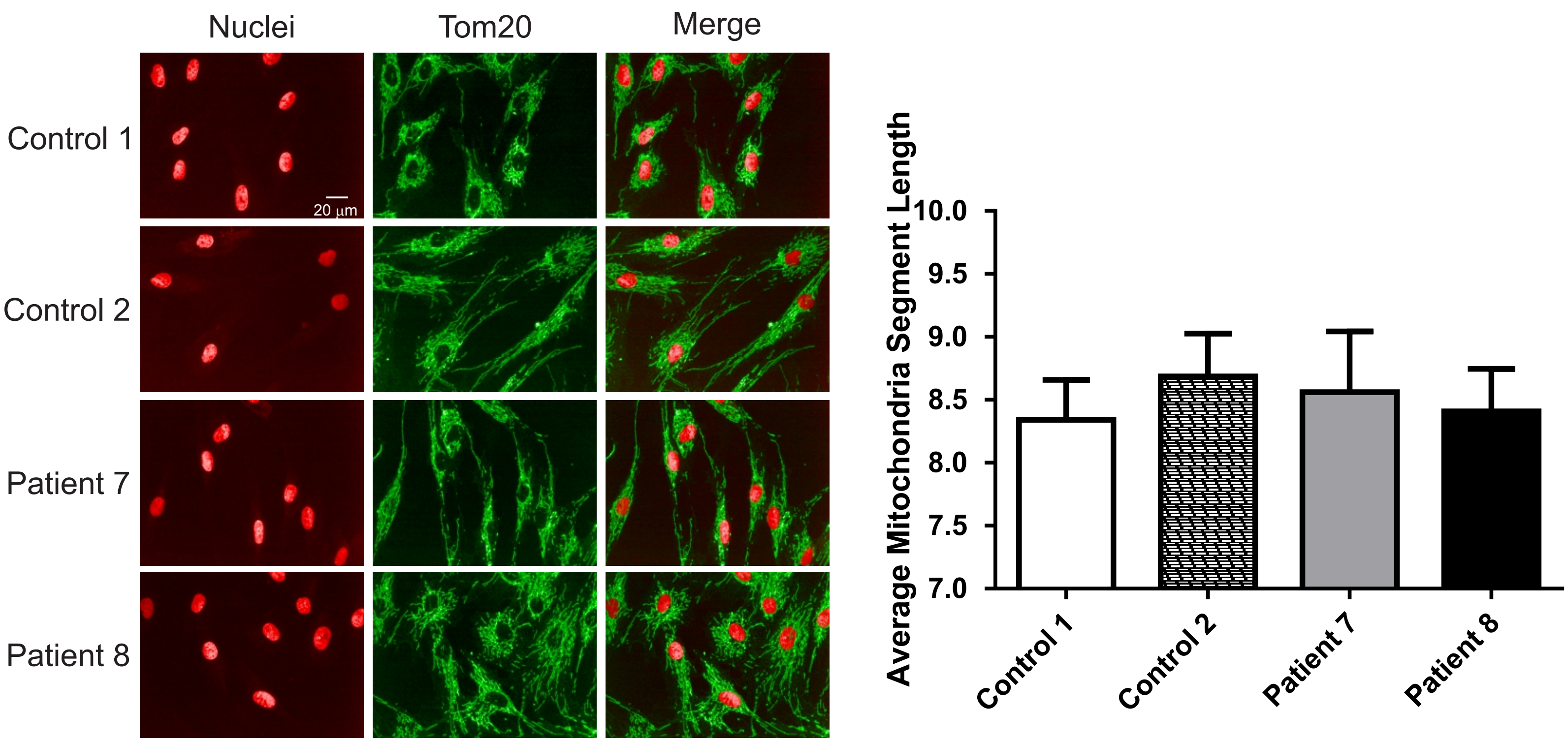


Supplemental Figure 1: Immunofluorescent imaging of mitochondrial network in fibroblast cells. **(Left)** Nuclei are identified by Hoechst stain (red) and mitochondria are stained with an anti-Tom20 antibody (green). Representative images are shown. **(Right)** The mitochondria segment length was calculated as the pixel distance from between either the end of a mitochondria or a branch point or the crossing over of another mitochondria and is averaged for each cell using a custom Acapella script run in the Columbus software. From 100 to 200 cells were measured per well and averaged over 14 wells for three separate experiments.

Supplemental Figure 2

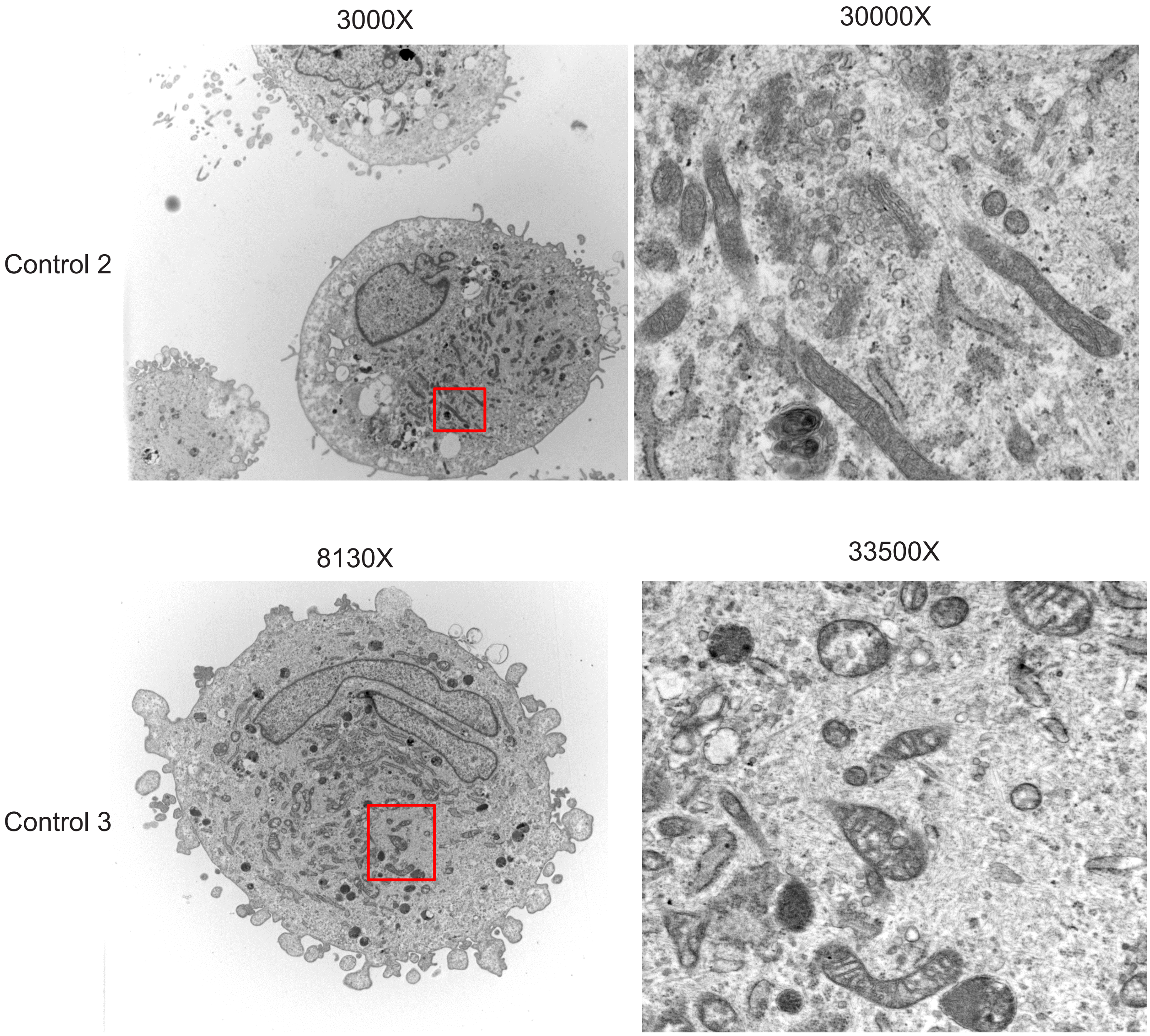


Supplemental Figure 2: Electron microscopy images of additional control fibroblasts. The red box identifies the area enlarged on the right.

Supplemental Figure 3

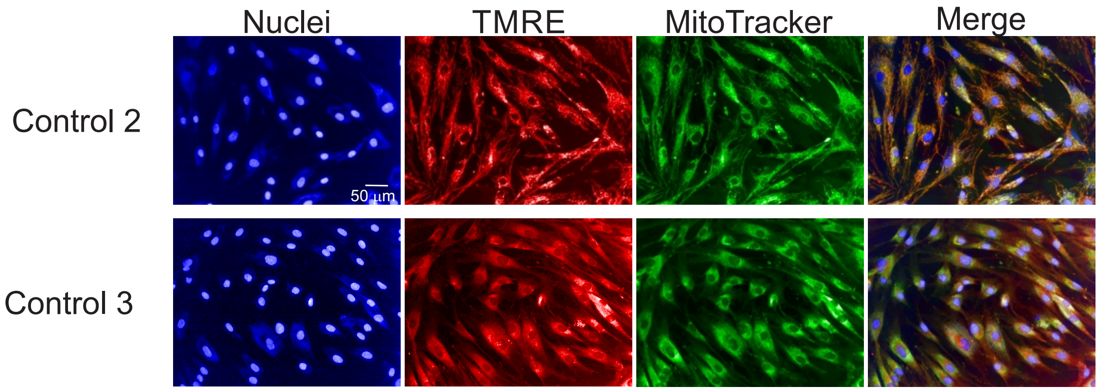


Supplemental Figure 3: Additional control fibroblasts for membrane potential assessment. Shown are immunofluorescence images of the mitochondrial membrane potential as monitored by the incorporation of the TMRE dye (red) and compared to the total mitochondrial stain as measured by MitoTracker (green). Nuclei are identified by Hoechst stain (blue).

Supplemental Figure 4

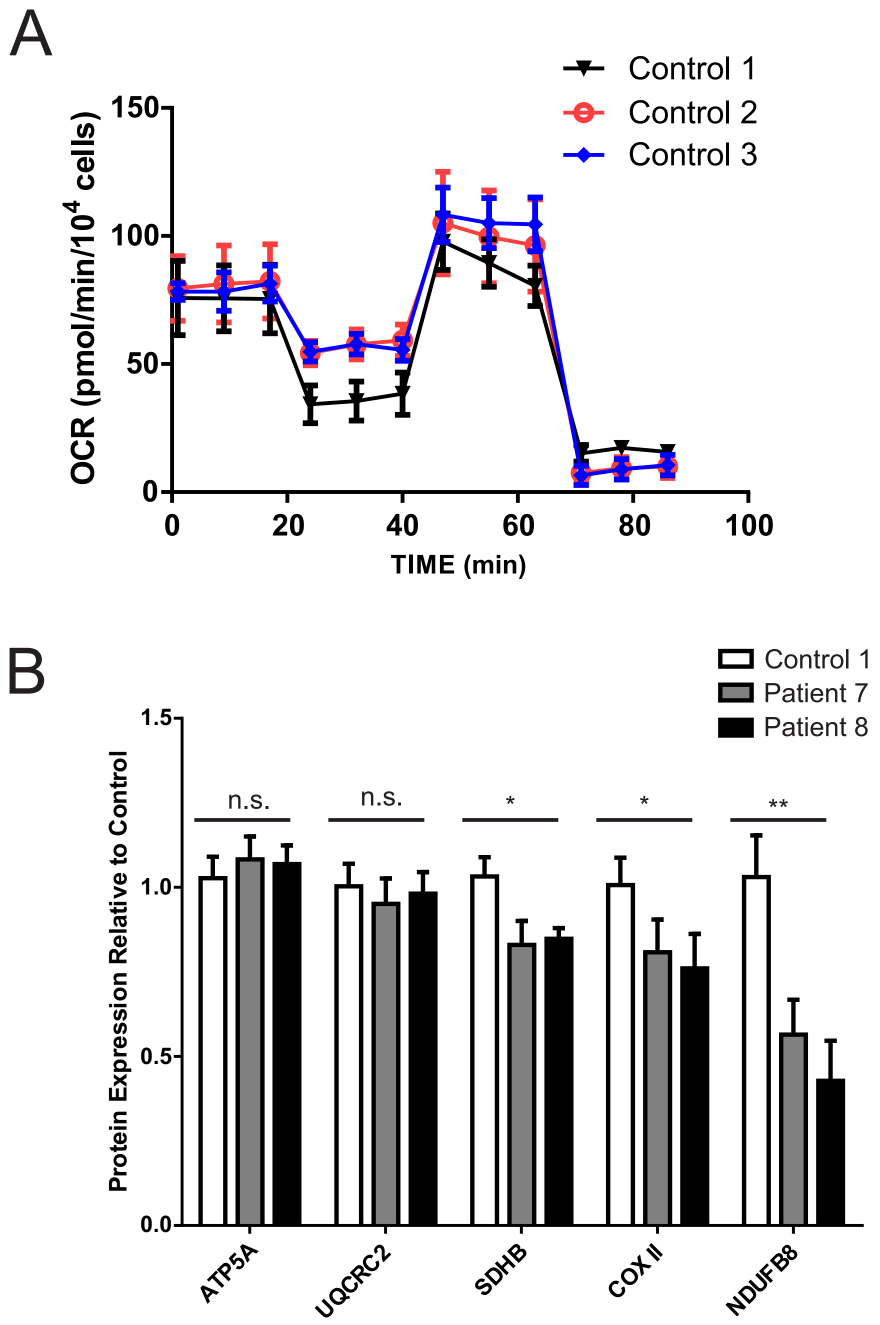


Supplemental Figure 4: (A) Oxygen consumption rate (OCR) of three control fibroblasts as measured by micro-oximetry analysis. (B) Densitometry of western blot analysis of 5 OXPHOS proteins. The band intensities were determined using ImageJ software. The intensity of each band is shown relative to total protein (ns = not significant; * = $p<0.05$; ** = $p<0.01$; student *t*-test).
